# Supplementary material for: Allosteric regulation and crystallographic fragment screening of SARS-CoV-2 NSP15 endoribonuclease
Source: Nucleic Acids Res. 2023 Apr 28;51(10):5255–70. doi: 10.1093/nar/gkad314 (PMC10250223; doi:10.1093/nar/gkad314)
Supplement: gkad314_Supplemental_Files [file gkad314_supplemental_files.zip › Supplementary datasets.zip!/188/Refine_9/01-BUSTER/Cycle-4/shell.01/R_Rfree.html]

# Rxpct factors in resolution bins at cycle 0


|  |  |  |  |  |  |  |  |  |
| --- | --- | --- | --- | --- | --- | --- | --- | --- |
|  | **<d> (Å)**  Rxpct\_Work | Rxpct\_Free | Rxpct\_All | |  |  |  |  |
|  | 16.0655 | 0.3903 | 0.4953 | 0.3938 |  |
|  | 9.2754 | 0.2216 | 0.2735 | 0.2246 |  |
|  | 7.1847 | 0.1957 | 0.2355 | 0.1980 |  |
|  | 6.0722 | 0.1971 | 0.2498 | 0.1994 |  |
|  | 5.3552 | 0.1570 | 0.2747 | 0.1618 |  |
|  | 4.8439 | 0.1462 | 0.2142 | 0.1502 |  |
|  | 4.4558 | 0.1420 | 0.2051 | 0.1452 |  |
|  | 4.1481 | 0.1489 | 0.2102 | 0.1525 |  |
|  | 3.8965 | 0.1567 | 0.2304 | 0.1602 |  |
|  | 3.6857 | 0.1668 | 0.2492 | 0.1705 |  |
|  | 3.5058 | 0.1727 | 0.2125 | 0.1747 |  |
|  | 3.3499 | 0.1771 | 0.2378 | 0.1806 |  |
|  | 3.2131 | 0.1912 | 0.2177 | 0.1927 |  |
|  | 3.0918 | 0.1910 | 0.2520 | 0.1938 |  |
|  | 2.9833 | 0.1933 | 0.2336 | 0.1950 |  |
|  | 2.8855 | 0.2044 | 0.2397 | 0.2062 |  |
|  | 2.7967 | 0.2043 | 0.2470 | 0.2062 |  |
|  | 2.7156 | 0.2110 | 0.2419 | 0.2125 |  |
|  | 2.6412 | 0.2619 | 0.2635 | 0.2620 |  |
|  | 2.5725 | 0.3467 | 0.3000 | 0.3451 |  |
|  | **Overall** | 0.1855 | 0.2414 | 0.1884 |  |

# Rxpct factors in resolution bins at cycle 1


|  |  |  |  |  |  |  |  |  |
| --- | --- | --- | --- | --- | --- | --- | --- | --- |
|  | **<d> (Å)**  Rxpct\_Work | Rxpct\_Free | Rxpct\_All | |  |  |  |  |
|  | 16.0655 | 0.3905 | 0.4949 | 0.3940 |  |
|  | 9.2754 | 0.2213 | 0.2741 | 0.2244 |  |
|  | 7.1847 | 0.1959 | 0.2355 | 0.1981 |  |
|  | 6.0722 | 0.1977 | 0.2517 | 0.2000 |  |
|  | 5.3552 | 0.1568 | 0.2758 | 0.1617 |  |
|  | 4.8439 | 0.1461 | 0.2139 | 0.1500 |  |
|  | 4.4558 | 0.1420 | 0.2056 | 0.1453 |  |
|  | 4.1481 | 0.1489 | 0.2105 | 0.1525 |  |
|  | 3.8965 | 0.1566 | 0.2306 | 0.1601 |  |
|  | 3.6857 | 0.1668 | 0.2494 | 0.1705 |  |
|  | 3.5058 | 0.1726 | 0.2126 | 0.1746 |  |
|  | 3.3499 | 0.1770 | 0.2379 | 0.1805 |  |
|  | 3.2131 | 0.1911 | 0.2176 | 0.1927 |  |
|  | 3.0918 | 0.1910 | 0.2520 | 0.1937 |  |
|  | 2.9833 | 0.1932 | 0.2334 | 0.1949 |  |
|  | 2.8855 | 0.2043 | 0.2396 | 0.2061 |  |
|  | 2.7967 | 0.2042 | 0.2471 | 0.2061 |  |
|  | 2.7156 | 0.2109 | 0.2418 | 0.2124 |  |
|  | 2.6412 | 0.2616 | 0.2636 | 0.2618 |  |
|  | 2.5725 | 0.3462 | 0.3010 | 0.3447 |  |
|  | **Overall** | 0.1855 | 0.2416 | 0.1883 |  |

# Rxpct factors in resolution bins at cycle 2


|  |  |  |  |  |  |  |  |  |
| --- | --- | --- | --- | --- | --- | --- | --- | --- |
|  | **<d> (Å)**  Rxpct\_Work | Rxpct\_Free | Rxpct\_All | |  |  |  |  |
|  | 16.0655 | 0.3906 | 0.4950 | 0.3940 |  |
|  | 9.2754 | 0.2213 | 0.2742 | 0.2244 |  |
|  | 7.1847 | 0.1959 | 0.2356 | 0.1982 |  |
|  | 6.0722 | 0.1977 | 0.2518 | 0.2001 |  |
|  | 5.3552 | 0.1567 | 0.2757 | 0.1616 |  |
|  | 4.8439 | 0.1460 | 0.2139 | 0.1500 |  |
|  | 4.4558 | 0.1420 | 0.2055 | 0.1453 |  |
|  | 4.1481 | 0.1489 | 0.2104 | 0.1524 |  |
|  | 3.8965 | 0.1565 | 0.2307 | 0.1600 |  |
|  | 3.6857 | 0.1667 | 0.2495 | 0.1704 |  |
|  | 3.5058 | 0.1725 | 0.2125 | 0.1745 |  |
|  | 3.3499 | 0.1770 | 0.2378 | 0.1805 |  |
|  | 3.2131 | 0.1911 | 0.2174 | 0.1926 |  |
|  | 3.0918 | 0.1909 | 0.2521 | 0.1937 |  |
|  | 2.9833 | 0.1932 | 0.2333 | 0.1949 |  |
|  | 2.8855 | 0.2042 | 0.2393 | 0.2060 |  |
|  | 2.7967 | 0.2042 | 0.2472 | 0.2061 |  |
|  | 2.7156 | 0.2108 | 0.2418 | 0.2123 |  |
|  | 2.6412 | 0.2616 | 0.2635 | 0.2617 |  |
|  | 2.5725 | 0.3458 | 0.2985 | 0.3443 |  |
|  | **Overall** | 0.1855 | 0.2416 | 0.1883 |  |

# Rxpct factors in resolution bins at cycle 3


|  |  |  |  |  |  |  |  |  |
| --- | --- | --- | --- | --- | --- | --- | --- | --- |
|  | **<d> (Å)**  Rxpct\_Work | Rxpct\_Free | Rxpct\_All | |  |  |  |  |
|  | 16.0655 | 0.3886 | 0.4879 | 0.3919 |  |
|  | 9.2754 | 0.2218 | 0.2769 | 0.2250 |  |
|  | 7.1847 | 0.1928 | 0.2367 | 0.1953 |  |
|  | 6.0722 | 0.1977 | 0.2577 | 0.2004 |  |
|  | 5.3552 | 0.1543 | 0.2722 | 0.1592 |  |
|  | 4.8439 | 0.1441 | 0.2216 | 0.1487 |  |
|  | 4.4558 | 0.1405 | 0.2031 | 0.1437 |  |
|  | 4.1481 | 0.1471 | 0.2087 | 0.1506 |  |
|  | 3.8965 | 0.1544 | 0.2355 | 0.1582 |  |
|  | 3.6857 | 0.1641 | 0.2534 | 0.1681 |  |
|  | 3.5058 | 0.1710 | 0.2095 | 0.1729 |  |
|  | 3.3499 | 0.1773 | 0.2366 | 0.1807 |  |
|  | 3.2131 | 0.1923 | 0.2192 | 0.1939 |  |
|  | 3.0918 | 0.1927 | 0.2644 | 0.1959 |  |
|  | 2.9833 | 0.1965 | 0.2361 | 0.1982 |  |
|  | 2.8855 | 0.2038 | 0.2435 | 0.2058 |  |
|  | 2.7967 | 0.2048 | 0.2578 | 0.2072 |  |
|  | 2.7156 | 0.2115 | 0.2482 | 0.2133 |  |
|  | 2.6412 | 0.2616 | 0.2610 | 0.2615 |  |
|  | 2.5725 | 0.3519 | 0.2685 | 0.3492 |  |
|  | **Overall** | 0.1846 | 0.2435 | 0.1876 |  |
